# Total Aortic Arch Replacement: Superior Ventriculo-Arterial Coupling with Decellularized Allografts Compared with Conventional Prostheses

**DOI:** 10.1371/journal.pone.0103588

**Published:** 2014-07-31

**Authors:** Alexander Weymann, Tamás Radovits, Bastian Schmack, Sevil Korkmaz, Shiliang Li, Nicole Chaimow, Ines Pätzold, Peter Moritz Becher, István Hartyánszky, Pál Soós, Gergő Merkely, Balázs Tamás Németh, Roland Istók, Gábor Veres, Béla Merkely, Konstantin Terytze, Matthias Karck, Gábor Szabó

**Affiliations:** 1 Department of Cardiac Surgery, Heart and Marfan Center, University of Heidelberg, Heidelberg, Germany; 2 Heart and Vascular Center, Semmelweis University, Budapest, Hungary; 3 Department of General and Interventional Cardiology, University Heart Center Hamburg Eppendorf, Hamburg, Germany; 4 2^nd^ Department of Pathology, Semmelweis University, Budapest, Hungary; 5 Federal Environment Agency, Dessau-Roβlau, Germany; 6 Department of Earth Science, Free University Berlin, Berlin, Germany; University of Louisville, United States of America

## Abstract

**Background:**

To date, no experimental or clinical study provides detailed analysis of vascular impedance changes after total aortic arch replacement. This study investigated ventriculoarterial coupling and vascular impedance after replacement of the aortic arch with conventional prostheses vs. decellularized allografts.

**Methods:**

After preparing decellularized aortic arch allografts, their mechanical, histological and biochemical properties were evaluated and compared to native aortic arches and conventional prostheses in vitro. In open-chest dogs, total aortic arch replacement was performed with conventional prostheses and compared to decellularized allografts (n = 5/group). Aortic flow and pressure were recorded continuously, left ventricular pressure-volume relations were measured by using a pressure-conductance catheter. From the hemodynamic variables end-systolic elastance (Ees), arterial elastance (Ea) and ventriculoarterial coupling were calculated. Characteristic impedance (Z) was assessed by Fourier analysis.

**Results:**

While Ees did not differ between the groups and over time (4.1±1.19 vs. 4.58±1.39 mmHg/mL and 3.21±0.97 vs. 3.96±1.16 mmHg/mL), Ea showed a higher increase in the prosthesis group (4.01±0.67 vs. 6.18±0.20 mmHg/mL, P<0.05) in comparison to decellularized allografts (5.03±0.35 vs. 5.99±1.09 mmHg/mL). This led to impaired ventriculoarterial coupling in the prosthesis group, while it remained unchanged in the allograft group (62.5±50.9 vs. 3.9±23.4%). Z showed a strong increasing tendency in the prosthesis group and it was markedly higher after replacement when compared to decellularized allografts (44.6±8.3dyn·sec·cm^−5^ vs. 32.4±2.0dyn·sec·cm^−5^, P<0.05).

**Conclusions:**

Total aortic arch replacement leads to contractility-afterload mismatch by means of increased impedance and invert ventriculoarterial coupling ratio after implantation of conventional prostheses. Implantation of decellularized allografts preserves vascular impedance thereby improving ventriculoarterial mechanoenergetics after aortic arch replacement.

## Introduction

Alexis Carrel, the pioneer of vascular surgery, was the first to describe the assets and drawbacks of autogenous and synthetic grafts. The first clinical applications of synthetic and biologic vascular grafts were performed in the 1950s [1],[2] and have become a standard treatment of aortic diseases [3]–[7].

Dacron (polyethylene terephthalate), for example, is a standard material used in aortic surgery acclaimed for its straightforward use and long lasting stability but has distinctly different mechanical properties than the native aorta. Several investigators demonstrated the variance in mechanical properties between native aortic tissue and prosthetic material leading to pressure and flow alterations in the vasculature [8]–[12]. Through prosthesis implantation and the associated flow changes, peripheral vascular diseases and the function of the aortic valve as well as the left ventricle can be negatively influenced [13], [14]. Furthermore, the developed compliance mismatch can contribute to local remodeling with abnormal wall shear stress on the border from native to prosthetic tissue [15], the consequence being the formation of a false aneurysm and/or graft thrombosis [16], [17]. Despite these known adverse effects, no data exist to date describing ventriculoarterial coupling and vascular impedance after replacement of the aortic arch with commonly used materials in reconstructive aortic arch surgery.

Furthermore, phthalates (component of Dacron) have been reported to evoke foreign-body reactions, to induce hepatic peroxisome proliferation and cancer, and adverse reproductive, developmental and endocrine effects [18], [19]. Ito et al. demonstrated increased thromboxane levels and decreased platelet counts one year after Dacron graft implantation in an animal model [20]. It is well known that deposition and activation of platelets evoke the thrombogenic nature of the synthetic graft with early and late graft failure.

Our group already reported in-vitro results, clinical experience with in-vivo-developed tissue-engineered pulmonary heart valves [21], [22] and creation of decellularized hearts as potential neoscaffolds for whole heart tissue engineering (TE) [23]. In this project, we were aimed at creating decellularized aortic arch allografts and analysing their biochemical composition and mechanical properties in vitro. Furthermore, we investigated for the first time decellularized aortic arch allografts implanted in an in-vivo model of total aortic arch replacement with hypothermic circulatory arrest and selective antegrade cerebral perfusion. To promote a deeper understanding of ventricular mechanoenergetics, we assessed ventricular and vascular properties by means of pressure-volume and impedance spectrum analysis in our experimental model of total aortic arch replacement.

## Methods

For a more detailed description of the methods, see Data S1.

### Preparation of Decellularized Aortic Arch Allografts

Canine aortic arches (n = 30) were harvested under sterile conditions from euthanized dogs (foxhounds) of other ongoing experimental studies. After the separation of adhesive tissue, all samples were examined macroscopically to exclude any pathology and stored in Medium 199 with Earle's salts (PAA Laboratories GmBH, Pasching, Austria) containing 10% dimethyl sulfoxide (DMSO) at -80°C until further use. Aortic arches were decellularized through continuous shaking in 1% sodium dodecyl sulfate (SDS) and 0.05% sodium azide (NaN_3_) in phosphate buffered saline (PBS) (PAA Laboratories) at room temperature for 48 h. The solution was exchanged every 6 h. At the end of the decellularization protocol, the aortic arches were washed with PBS for 12 h to remove residual detergents and cell debris, then stored in 1% penicillin-streptomycin (PAA Laboratories, Cölbe, Germany) augmented Earle's Medium 199 (PAA Laboratories) until implantation/other measurements (Figure S1, left side).

### DNA Quantification

DNA was isolated from 200-mg freeze-dried, decellularized aortic arch allografts and processed for spectrophotometric quantification to determine the concentration of residual DNA in the decellularized group in comparison with the native control group. The total amount of DNA was purified using a silica-membrane based method, following the manufacturer's instructions, and later quantified by spectrophotometry (QIAamp DNA Mini Kit, Qiagen, Basel, Switzerland).

### Histological Analysis

Segments of decellularized aortic arch allografts and freshly harvested aortic arches (control) underwent standard histological processing followed by hematoxylin-eosin (HE), Masson's trichrome and Movat's pentachrome staining to visualize tissue structure and extracellular matrix (ECM) components (see Data S1).

### Transmission Electron Microscopy (TEM)

Aortic arch specimens (1 mm^3^) were fixed in 4% glutaraldehyde (EMD Chemicals Inc, Gibbstown, NJ, USA) and 0.1 M sodium cacodylate trihydrate (Sigma-Aldrich, Germany). Fixed segments were ultrathin-sectioned and prepared according to standard procedure. Electron microscopy was performed using a FEI Tenmai Biotwin transmission electron microscope.

### Quantification of Collagen and Elastin Content

To quantify collagen and elastin, Biocolor assays (Biocolor, Carrickfergus, UK) were performed in decellularized aortic arch samples and compared with native tissue. Collagen was extracted with 0.5 mol/L acetic acid (Sigma-Aldrich) and 1∶50,000 protease inhibitor cocktail at 4°C. Elastin was extracted with 100°C 0.25 mol/L oxalic acid (Sigma-Aldrich). Samples and calibrators were treated with the respective dyes and later quantified by spectrophotometry.

### Measurement of Mechanical Stability

Mechanical stability of conventional prostheses and decellularized allografts was analyzed using a static material testing instrument (Zwick Roell, Ulm, Germany) and compared to native, untreated controls of aortic arch tissue. In order to better simulate in vivo conditions, prostheses, native aortic arches and decellularized allografts were first placed under a resting tension of 0.05N (3 times), which ensured a proper “prepressurization”. Afterwards, conventional prostheses (Dacron), decellularized allograft samples and untreated controls were stretched until complete tearing in longitudinal and circumferential directions. Passive tensile strength was constantly registered during the displacement.

### Experimental Model of Total Aortic Arch Replacement

#### Animals, Ethics Statement

10 dogs (foxhounds, WOBE Kft., Budapest, Hungary) of both sexes weighing 24.5 to 35 kg (30.1±1.0 kg) were used in these experiments. All animals received human care in compliance with the ”Principles of Laboratory Animal Care” formulated by the National Society for Medical Research and the ”Guide for the Care and Use of Laboratory Animals” prepared by the Institute of Laboratory Animal Resources and published by the National Institutes of Health (NIH Publication No. 86-23, revised 1996). The Scientific Ethical Committee of Hungary for Animal Experimentation approved the experiments (permit Nr.: 22.1/1163/3/2010). All surgery was performed under proper anesthesia, and all efforts were made to minimize suffering.

#### General Management and Surgical Preparation

The dogs were anesthetized, endotracheally intubated and ventilated. The right femoral vein and artery were cannulated for volume substitution and for taking blood samples. Arterial pressure was monitored with 6F Millar pressure catheter inserted into the abdominal aorta via the left femoral artery (Millar Instruments, Houston, TX, USA) (see Data S1).

#### Total Aortic Arch Replacement

After left anterolateral thoracotomy in the fourth intercostal space and pericardiotomy, the great vessels including the aortic arch with the bilateral innominate arteries were exposed. After systemic anticoagulation with sodium heparin (300 U/kg), the left subclavian artery was cannulated for arterial perfusion. The venous cannula was placed in the right atrium. The extracorporeal circuit consisted of a heat exchanger, a venous reservoir, a roller pump, and a membrane oxygenator primed with Ringer lactate solution (1000 ml) supplemented with heparin (150 U/kg) and 20 ml sodium bicarbonate (8.4%). After injection of prednisolon (250 mg i.v.) and initiation of cardiopulmonary bypass (CPB), cooling of the body temperature was started and the pump flow was set to 100 ml/kg/min to maintain perfusion pressure above a value of 35-40 mmHg at any time point. After crossclamping of the aorta, the heart was arrested with 10 ml/kg cold (4°C) HTK solution (Custodiol, Dr. Franz Köhler Chemie GmBH, Alsbach-Hähnlein, Germany).

After induction of cardiac arrest, and obtaining systemic hypothermia (body temperature at 24.3±0.6°C) CPB was stopped and the left carotid artery was cannulated for selective antegrade cerebral perfusion (hypothermic oxygenized blood perfusion, pump flow at 10 ml/kg/min, duration: 22.2±1.7 min).

The aortic arch of the animal was removed and replaced by a size-matched (8 mm in diameter and 50 mm in length) conventional Dacron prosthesis (prosthesis group, n = 5) or decellularized allograft (allograft group, n = 5) (Figure S1, right side). After completion of each anastomosis the aorta was declamped, reperfusion was started and rewarming was initiated. The mean duration of circulatory arrest was 45.6±4.3 minutes. If necessary, ventricular fibrillation was counteracted with DC cardioversion of 20J. Ventilation was restarted with 100% oxygen during reperfusion and weaning from CPB. All animals were weaned from CPB 60 min after the release of the aortic cross clamp.

#### Hemodynamic Measurements and Analysis

Hemodynamic measurements were performed at baseline (before starting CPB) and after total aortic arch replacement (15 min after weaning from CPB).

Heart rate (HR), mean arterial pressure (MAP), left ventricular (LV) pressures and volumes, cardiac output (CO), cardiac index (CI), stroke volume (SV), the time constant of LV pressure decay (τ), total peripheral resistance (TPR), total peripheral resistance index (TPRI), stroke work (SW), stroke work index (SWI), pressure-volume area (PVA), vascular impedance spectrum, input impedance (RIN) and characteristic impedance (Z) were determined by arterial pressure- and LV pressure-volume (P-V) analysis [24], [25] and by Fourier analysis of recorded pressure and flow data [26], [27] (see Data S1). Arterial elastance (Ea) was calculated as the quotient of end-systolic pressure (Pes) and SV (Ea = Pes/SV). End-systolic elastance (Ees) was determined as the slope of the LV end-systolic P-V relation. Ventriculoarterial coupling (VAC) was described by the quotient of Ea and Ees (VAC = Ea/Ees). The SW/PVA ratio was defined as mechanical efficiency (Eff).

### Statistical Methods

All data are expressed as means±SEM. Statistical analysis was performed on a personal computer with the Origin 7G software. A paired t-test was used to compare two means within a group (comparison of “baseline” and “after replacement” values). Means between the groups were compared by an unpaired two-sided Student's t-test (comparison of prosthesis group and allograft group). A p-value less than 0.05 was considered statistically significant.

## Results

### Histological and Ultrastructural Analysis of Tissue Morphology

**Figure 1:**
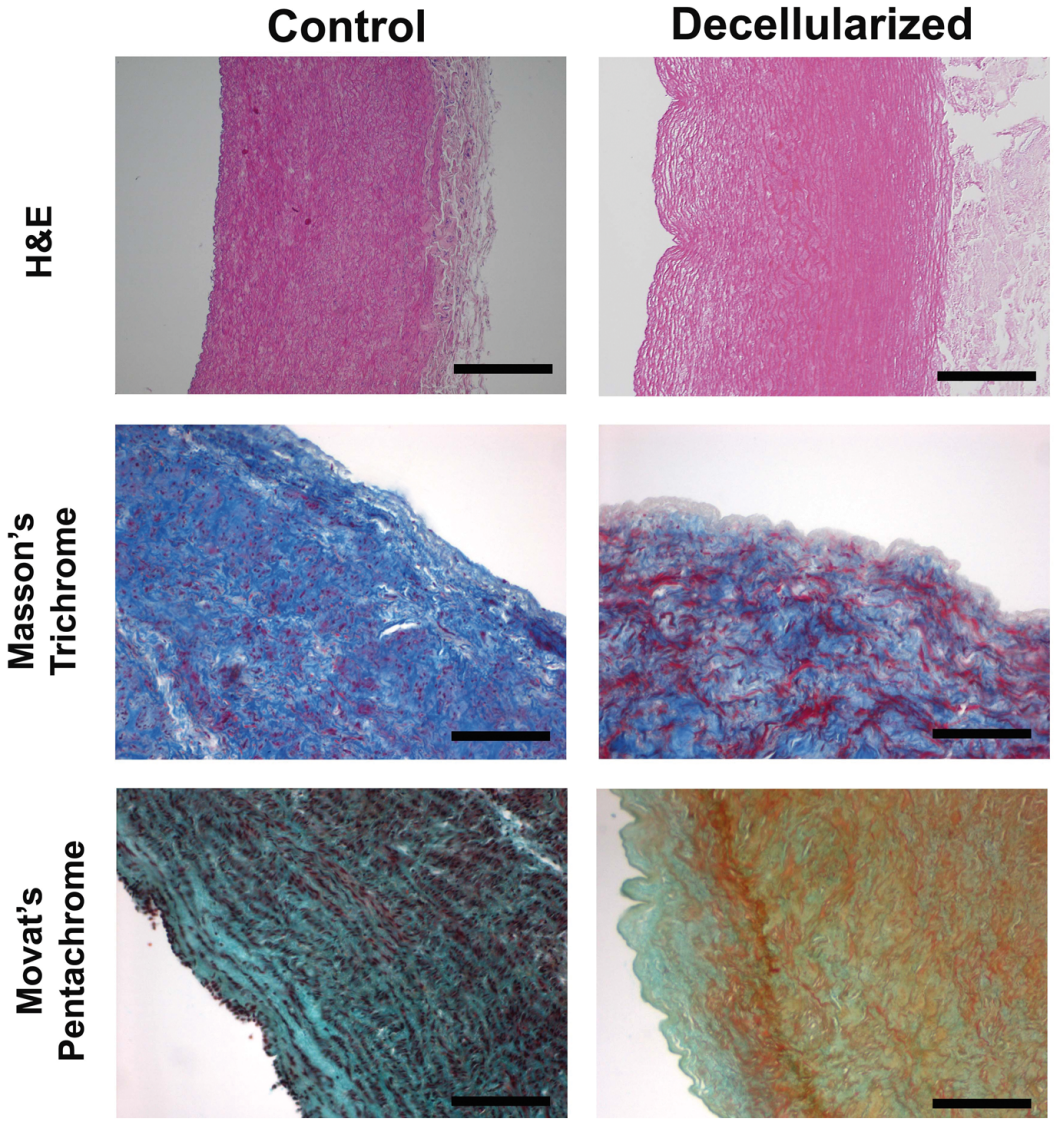
ECM composition of aortic arch samples. HE stain, Masson's Trichrome stain and Movat's Pentachrome stain show complete preservation of the ECM after the decellularization procedure of aortic arch specimens. Bars, 50 µm. Masson's Trichrome stain: cytoplasm (red), collagen (blue), nuclei (dark brown). Movat's Pentachrome stain: nuclei (dark purple to black), elastic fibres (purple to black), collagen (yellow), glycosaminoglycans (green), mucin (blue), cytoplasm (pink to brownish-red).

The decellularized aortic arch specimens demonstrated stability of the extracellular matrix, while the cellular components and the endothelial cell layer were completely removed, as shown by standard histology and TEM. HE staining demonstrated the overall structure intact after decellularization. Furthermore, Masson's Trichrome and Movat's Pentachrome stain visualized an optimally preserved 3-dimensional neoscaffold composition with different ECM elements as for instance the typical mesh-like collagen structures, elastin and proteoglycans (Fig. 1).

**Figure 2:**
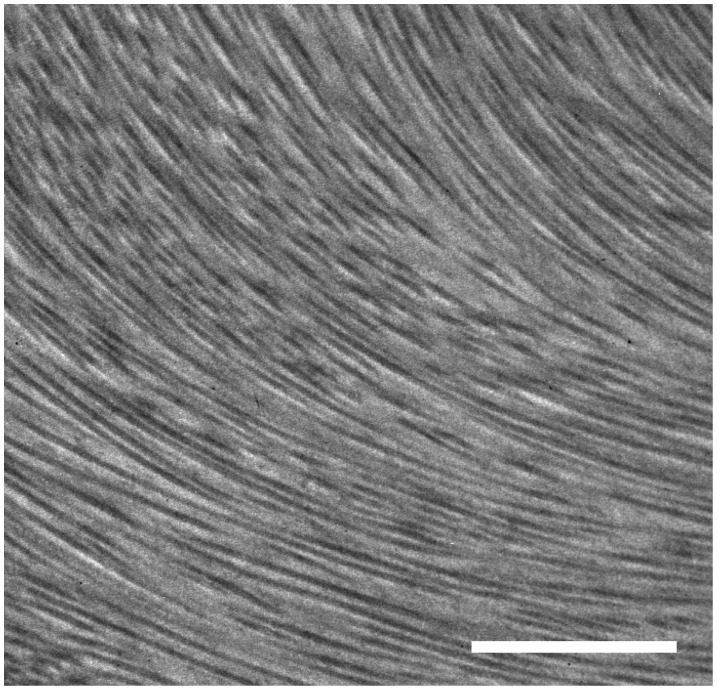
Transmission electron microscopy. Transmission electron microscopy demonstrating retained collagen fibrils after decellularization treatment. Bar, 2 µm.

The analytical TEM investigation depicted collagen-fiber bundle networks after the decellularization process without evidence of nuclear material (Fig. 2).

**Figure 3:**
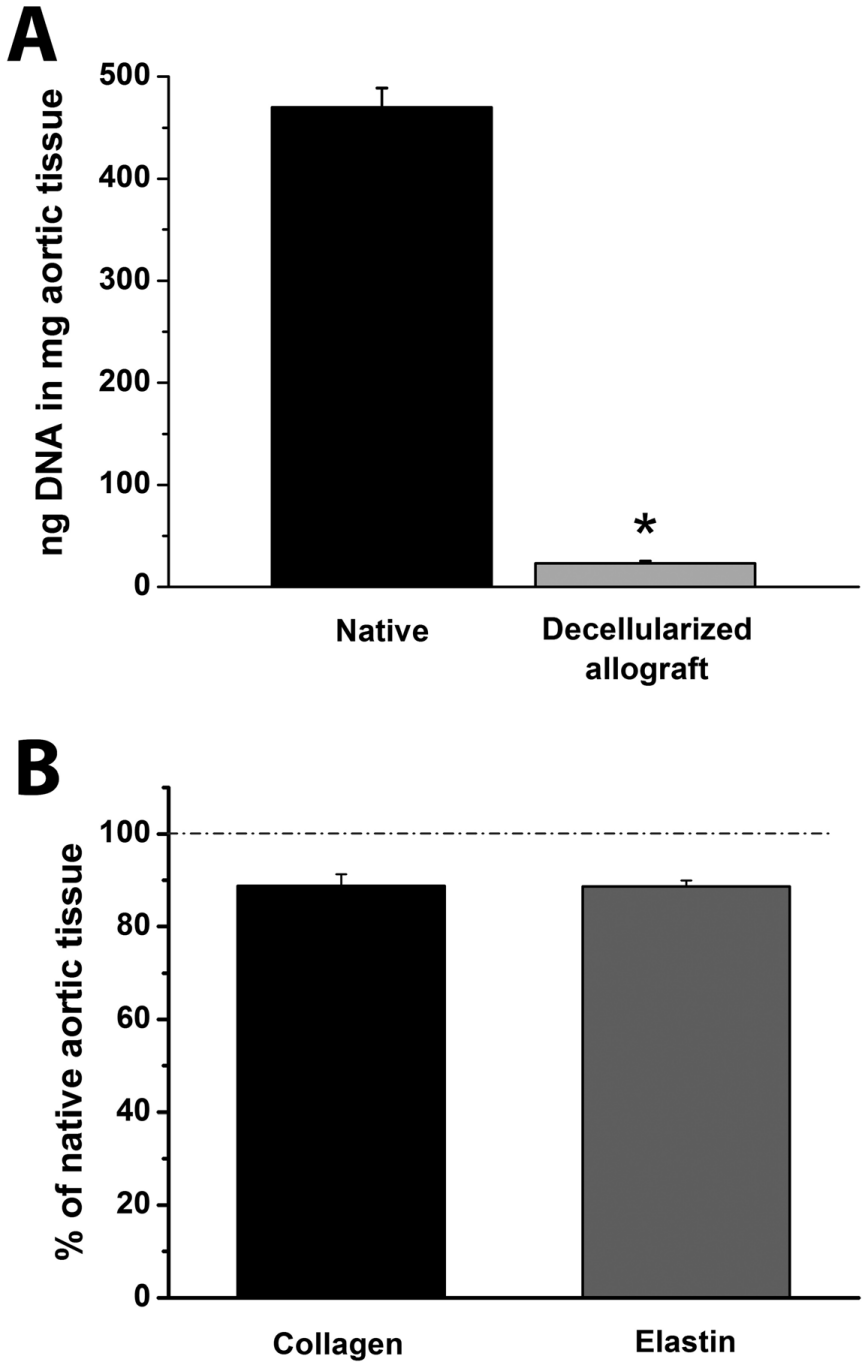
DNA, collagen and elastin content. Total DNA content of native aortic arch tissue and decellularized aortic arch allografts (A). Collagen and elastin content of decellularized aortic arch allograft samples compared to that of native aortic arch tissue (B) *:P<0.05 vs. Native aortic arch

The results noted above, were confirmed by quantitative evaluation of DNA content. After decellularization, the DNA content of aortic arch tissue was significantly reduced (under the level of 5%) (Fig. 3 A).

In summary, intracellular material was successfully removed from native aortic arch specimens, while the natural ECM composition was preserved.

### Quantitative Analysis of Collagen and Elastin Content

Collagen and elastin content of decellularized aortic arch samples was compared with that of untreated aortic arch tissue, which was taken as 100% (Fig. 3 B). Quantification of collagen and elastin content revealed 88.76±2.52% and 88.66±1.25%, respectively, compared with native tissue. Hence, there was no significant change in collagen and elastin content after decellularization.

### Analysis of Mechanical Properties

**Figure 4:**
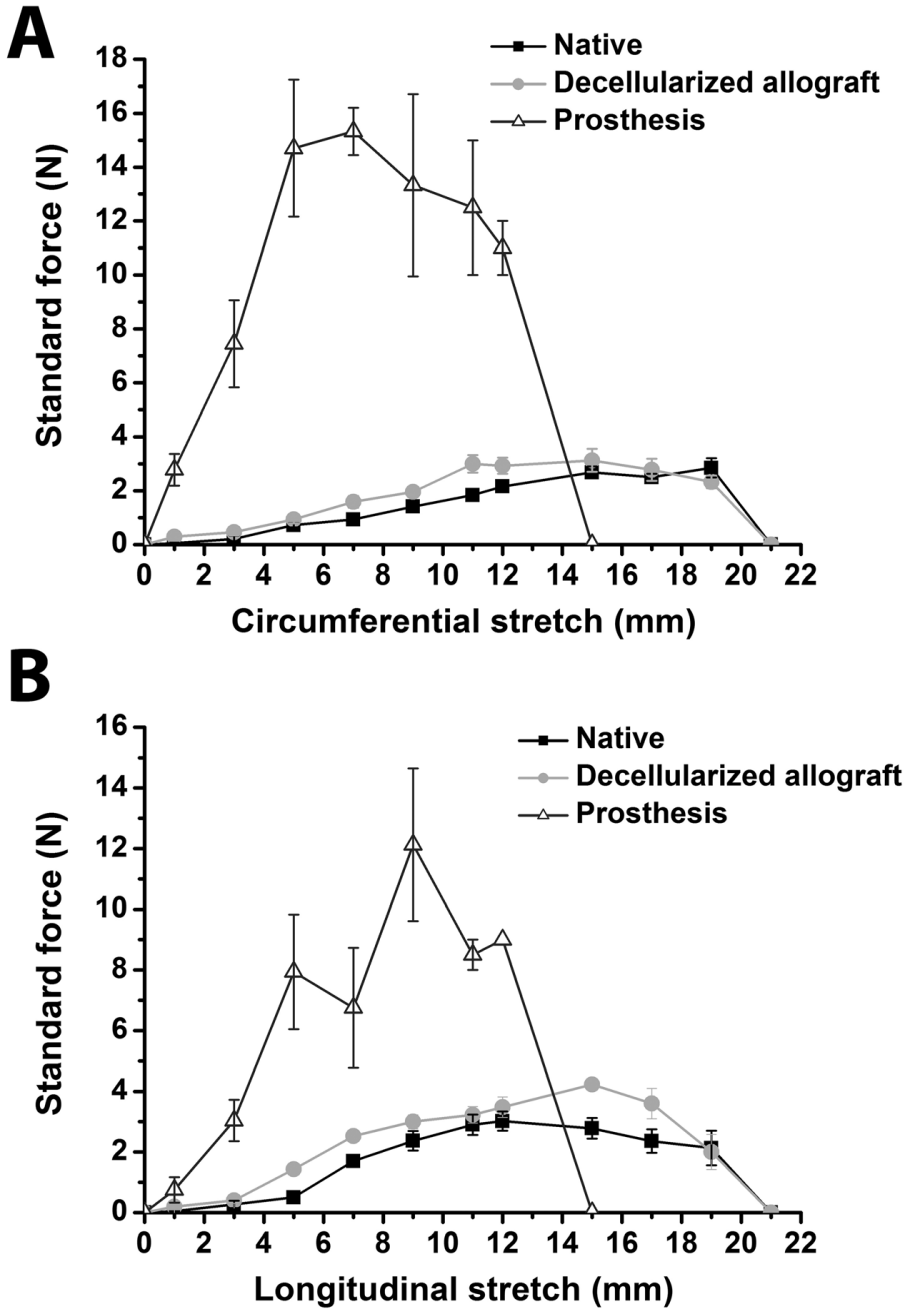
In vitro biomechanical properties. Circumferential (A) and longitudinal (B) stress-strain curves of prostheses, native and decellularized aortic arches. All data are expressed as means ± SEM.

In order to assess the effects of decellularization on the elastic behaviour, equi-biaxial tensile tests were performed on aortic arch tissue before and after decellularization and compared with conventional prosthetic material. Results of tensile viscoelastic properties are shown in Fig. 4. Native aortic arch samples and decellularized aortic allografts almost showed no major differences for longitudinal and circumferential stretch, indicating an intact mechanical stability with similar anisotropic elastic responses. In comparison, the stress-strain curve of the conventional prosthesis group shows a severely abnormal behaviour, which is demonstrated by large changes in stress-strain and stiffness.

### Hemodynamics

Basic hemodynamic variables are shown in Table 1. Baseline values were within the physiological range and no significant difference could be documented between the groups. After aortic arch replacement, most of the hemodynamic parameters showed only marginal changes. CO, CI, end-systolic (Pes) and end-diastolic pressure (Ped) and τ were nearly identical to baseline values in both groups. In contrast, a significantly decreased MAP and increased HR could be observed after aortic arch replacement. SV, SW, PVA and SWI showed only a decreasing tendency, without reaching the level of significance. A strong tendency towards decreased values of TPR and TPRI could be observed in the prosthesis group after aortic arch replacement, reaching the level of statistical significance in the allograft group.

**Table 1:** Basic hemodynamic parameters.

	Prosthesis		Decellularized allograft	
	Baseline	After replacement	Baseline	After replacement
HR (1/min)	113±3	146±6*	120±5	147±8*
MAP (mmHg)	71±4	41±2*	77±4	47±2*
CO (l/min)	2.50±0.48	2.24±0.15	2.15±0.18	2.22±0.27
CI (ml/min/kgBW)	79.9±15.4	71.5±5.1	77.3±7.2	78.5±8.2
SV (ml)	22.1±3.9	15.3±0.7	18.1±1.7	15.5±2.5
Pes (mmHg)	81±4	90±5	89±4	84±8
Ped (mmHg)	7.7±0.3	7.7±0.8	6.5±0.5	6.8±1.5
τ (ms)	33.2±2.5	28.3±4.2	30.6±1.1	29.5±5.3
TPR (mmHg/l/min)	31.1±5.1	18.3±1.1	39.0±2.6	22.9±3.5*
TPRI (mmHg/l/min/kgBW)	0.99±0.16	0.59±0.04	1.40±0.09	0.84±0.16*
SW (mmHg·ml)	1641±320	1334±132	1521±203	1188±205
SWI (mmHg·ml/kgBW)	52.5±10.4	42.7±4.6	55.1±8.3	42.3±7.3
PVA (mmHg·ml)	2533±379	2484±412	3145±586	2222±347

Hemodynamic parameters in both groups at baseline and after total aortic arch replacement. Values of heart rate (HR), mean arterial pressure (MAP), cardiac output (CO), cardiac index (CI), stroke volume (SV), left ventricular end-systolic (Pes) and end-diastolic pressure (Ped), time constant of left-ventricular pressure decay (τ), total peripheral resistance (TPR), total peripheral resistance index (TPRI), stroke work (SW), stroke work index (SWI) and pressure-volume area (PVA) are shown as mean±SEM. *:p<0.05 vs. baseline.

**Figure 5:**
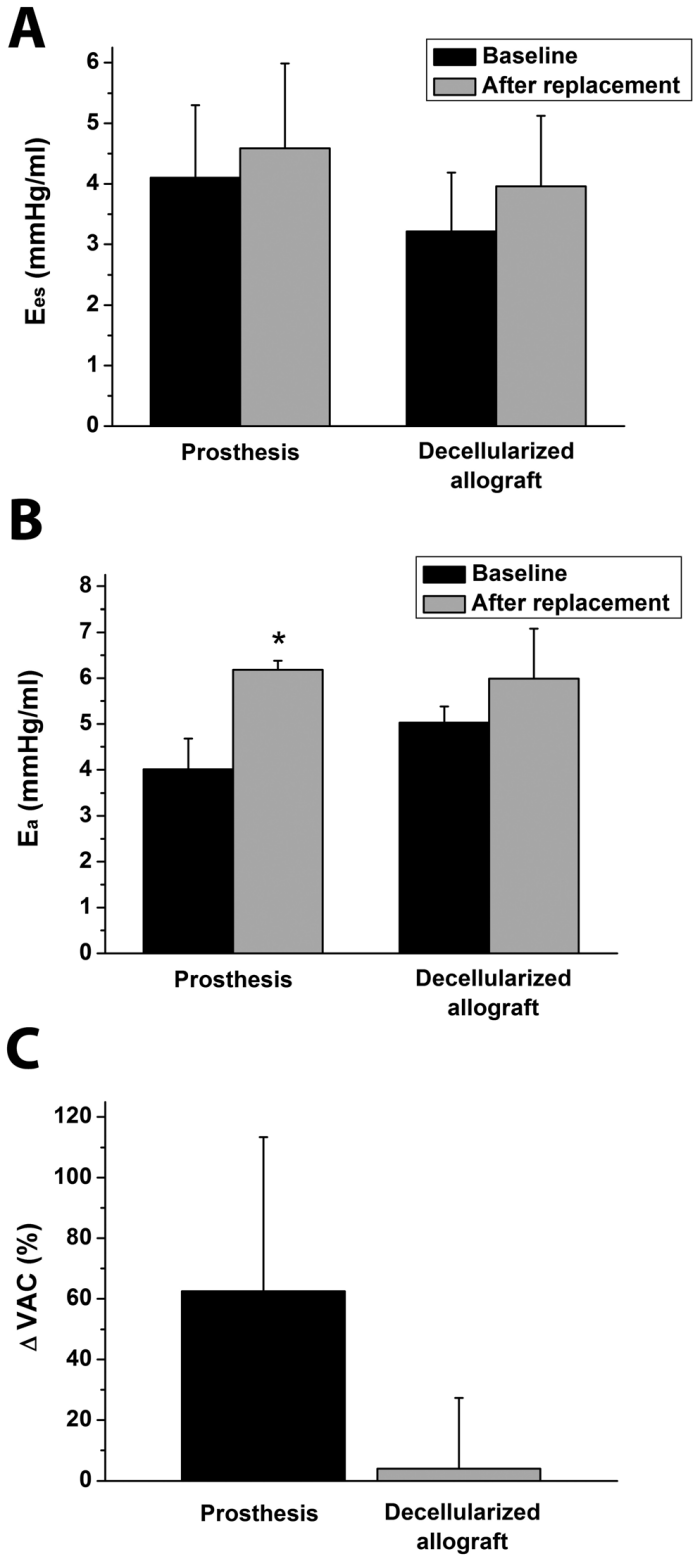
Contractility, afterload and ventriculoarterial coupling. End-systolic elastance (Ees, A), arterial elastance (Ea, B) at baseline and after total aortic arch replacement; and relative changes of ventriculoarterial coupling (VAC, C) in the prosthesis and decellularized allograft groups. All values are given as means ± SEM, *:P<0.05 vs. Baseline

LV P-V analysis revealed an unchanged Ees along with a significantly increased Ea in the prosthesis group after replacement. In contrast, both Ees and Ea remained unaltered in the allograft group (Fig. 5).

Correspondingly, VAC ratio (Ea/Ees) showed a marked increase in the prosthesis group and was nearly identical to baseline values in case of allografts (Fig. 5). Accordingly, Eff decreased in the prosthesis group (ΔEff: -17.4±11%) and remained unchanged in the allograft group (+6.5±10.3%) after replacement.

**Figure 6:**
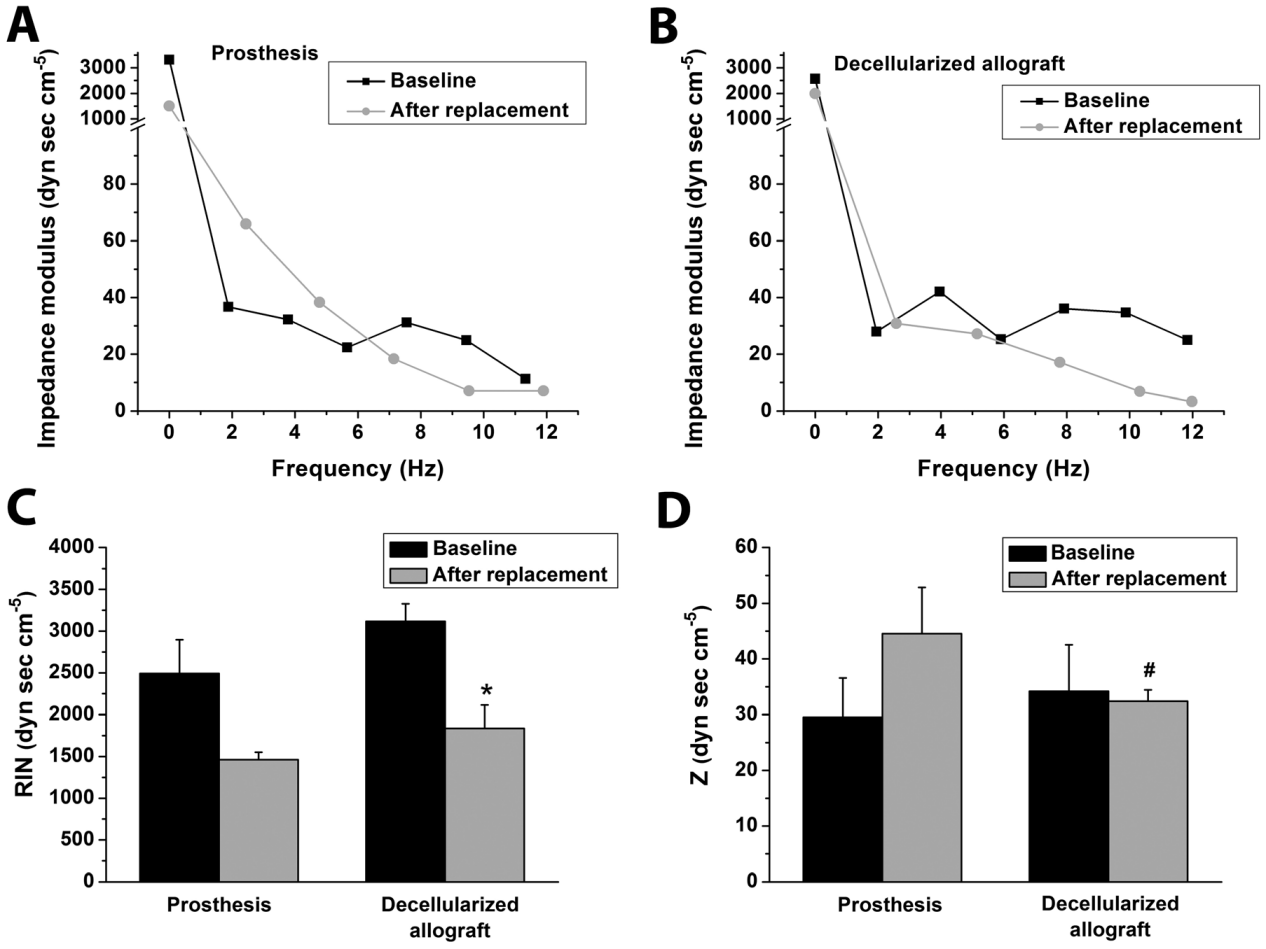
Vascular impedance spectrum. Vascular impedance spectrum after total aortic arch replacement in a representative animal of the prosthesis (A) and decellularized allograft group (B). Input impedance (RIN, C), and characteristic impedance (Z, D) at baseline and after total aortic arch replacement in both groups. All values on panels C and D are given as means ± SEM, *:P<0.05 vs. Baseline, #:P<0.05 vs. Prosthesis

Fourier analysis of impedance spectrums showed a decrease of RIN after replacement. A strong tendency towards increased Z values has been observed in the prosthesis group after replacement, while it remained unchanged in the decellularized allograft group. Z was significantly higher in the prosthesis group after replacement (Fig. 6). Representative impedance spectra from both groups are depicted in Fig. 6.

## Discussion

To our knowledge, this is the first experimental study about analysis of changes in vascular impedance after total aortic arch replacement. Moreover, we describe for the first time a successful application of an in-vivo model of total aortic arch replacement with hypothermic circulatory arrest and selective antegrade cerebral perfusion.

Driven by the desire to develop an ideal vascular substitute, the present study provides in-depth knowledge of ventriculoarterial coupling and vascular impedance after replacement of the aortic arch with conventional prostheses vs. decellularized allografts. Our study showed that total aortic arch replacement leads to contractility-afterload mismatch by means of increased characteristic impedance and invert ventriculoarterial coupling ratio after implantation of a conventional prosthesis. Implantation of decellularized allografts preserved vascular impedance spectrum and thereby improved ventriculoarterial mechanoenergetics after aortic arch replacement.

### Effects of Decellularization Treatment on Matrix Structure

In this study, we used decellularized allografts and conventional prostheses to reconstruct the aortic arch. We were able to show, that the structural properties of the decellularized allografts were sustained while all cellular and nuclear material was efficiently removed. We already demonstrated successful cell elimination with SDS/NaN_3_ treatment [22] and in this investigation for the first time for aortic arch allografts. Transmission electron microscopy and histology studies were used to confirm the removal of cells and to investigate the composition and structure of tissue samples. Furthermore, the collagen and elastin content of the decellularized neoscaffolds demonstrated similar characteristics to untreated controls. We maintained the three-dimensional matrix and important structural proteins like collagen, elastin, and proteoglycans, parts missing in synthetic materials.

Synthetic-based scaffolds are rigid and potentially immunogenic, and additionally suffer from toxic degradation, induce an overshooting fibrosis and inflammatory reaction. Moreover, synthetic grafts cannot express important bioactive molecules and ligands, which are necessary for vessel maturation [21]–[23].

Decellularized allografts are appealing because they are already composed of native vascular extracellular matrix proteins that exhibit reasonable structural characteristics as well as providing instructive cues for cellular ingrowth. It was shown that bone marrow-derived cells incubated on decellularized canine carotid arteries, demonstrated cellular incorporation into the scaffold and subsequent differentiation into endothelial and vascular smooth muscle cells with three distinct vessel layers [28].

Taken together, these findings are favorable for recellularization of decellularized allografts once implanted in vivo as our group already demonstrated it for decellularized pulmonary heart valves in human subjects [21].

### Effects of Decellularization Treatment on Biomechanical Matrix Properties

Planar biaxial tensile test in both perpendicular directions has been used in this study to determine the mechanical behaviour of the applied tissues for aortic arch reconstruction.

It was already shown that replacement of aortic tissue by synthetic grafts reduces elasticity and limits the redistribution of energy from systole to diastole [29]. Other investigators described compliance differences induced by synthetic material used in aortic surgery, which caused a flow interruption in vivo and anastomotic neointimal hyperplasia [16].

We depicted in this investigation anisotropic elasticity behaviour of decellularized allografts similar to intact untreated aorta. Additionally, we could underline the significant negative differences in mechanical properties and behavior of synthetic material, which was demonstrated by large changes in stress-strain and stiffness, in comparison with native and decellularized aortic tissue.

### Hemodynamics and Vascular Impedance Analysis

We performed LV P-V and vascular impedance spectrum analysis to characterize mechanoenergetic changes after aortic arch replacement. We determined Ees and Ea, which are load-independent indices of ventricular contractility and vascular loading, respectively [30]. The alterations of many basic hemodynamic parameters after total aortic arch replacement were rather small, the only significant changes have been observed in the case of TPR/TPRI, subsequently MAP, all of which can be attributed to the common systemic reaction after CPB along with peripheral vasodilatation. Nevertheless, we report here for the first time that the observed hemodynamic changes after total aortic arch replacement with conventional prostheses have a profound influence on mechanoenergetics. The unaltered myocardial contractility (Ees) and the significant increase of Ea led to a marked worsening of the ventriculoarterial coupling ratio in the prosthesis group.

The data of the present study suggest that multiple (and in certain cases small) changes of afterload, preload, and left ventricular contractility additively result in unfavorable mechanoenergetics, which is in accordance with previous works [25]. We determined ventricular afterload in terms of TPR and Ea, as well as RIN and Z. Although TPR and RIN showed a tendency towards (prosthesis) or significantly lower values (decellularized allograft), this alteration only characterizes the state of peripheral precapillary resistence arteries, thus it can be unequivocally attributed to the CPR-induced peripheral vasodilatation. Because flow through the cardiovascular system is pulsatile, these conventional parameters of afterload exclude the significant contribution of pulsatile blood flow to the understanding of systemic hemodynamics. Moreover, the important function of large elastic arteries (Windkessel function) must also be taken into consideration in the aortic arch replacement setting. To further elucidate these aspects of afterload changes, we performed a Fourier analysis for assessment of vascular impedance spectrums distal to the aortic arch (Fig. 6). Although RIN (zero harmonics, an equivalent of TPR) showed decreased values after replacement, vascular impedance at harmonics between 1 and 6Hz was markedly increased in the prosthesis group, which is in line with previous studies [11]. This indicates an increased stiffness of the central arterial system and partial loss of the aortic Windkessel function and can be attributed to the synthetic Dacron material with strongly limited elastic properties compared to the native aortic arch. Impaired Windkessel properties increase wall tension and rate of pressure rise, which may have clinical impact with respect to a sudden and sustained rise of mechanical load in the residual aorta, especially at the vulnerable proximal descending part [10]. Moreover, impaired Windkessel function of the aorta has been proven to induce hypertrophy of the left ventricle and might lead to the development of heart failure [13], [14].

In contrast, replacement of the aortic arch with decellularized allografts was associated with unchanged Ees, Ea and VAC, indicating intact mechanoenergetics. Analysis of vascular impedance spectrum in the allograft group revealed completely unaltered, physiological stiffness and elastic properties of the arterial system with the implanted decellularized aortic arch allograft.

### Study Limitations

The present proof-of-concept study investigated only the acute functional aspects of total aortic arch replacement with decellularized allografts. Whether or not a responsive, self-renewing tissue graft with normal physiological functions can develop from the implanted decellularized aortic arch neoscaffold in the longer term, has to be evaluated in future chronic studies. Moreover, our study is based on an acute model, and the elastic properties might change over time due to formation of adhesions and scar.

### Conclusions

There are several key findings of this study. First, to the best of our knowledge, this is the first report of the generation of decellularized aortic arch allografts containing a preserved ECM composition. Second, we describe for the first time a successful application of an in-vivo model of total aortic arch replacement with hypothermic circulatory arrest and selective antegrade cerebral perfusion [31]. Third, total aortic arch replacement leads to contractility-afterload mismatch by means of increased impedance and invert ventriculoarterial coupling ratio along with impaired ventricular efficiency after implantation of a conventional prosthesis in our animal model. Implantation of decellularized allografts preserved characteristic impedance and ventricular efficiency, thereby improved ventriculoarterial mechanoenergetics after aortic arch replacement. Fourth, the fabricated aortic arch neoscaffold matches the elastic and viscoelastic properties of untreated aortic tissue.

In summary, these studies serve as proof of concept to generate bioengineered aortic arch neoscaffolds as an off-the-shelf alternative over currently available synthetic grafts. The decellularized allograft could be tailored to a range of lengths and diameters, widely available, and easily transported. Our work has the potential for an important clinical contribution, which is a strong argument for evaluating our approach experimentally in additionally studies with special reference to future clinical application.

## Supporting Information

Data S1 **Supplementary methods.** Detailed description of the methods used in the study.
(DOC)

Figure S1 **Photographs of the prosthesis and decellularized allografts.** Representative images of macroscopic appearance of an implanted conventional prosthesis (left panel), decellularized aortic arch allograft before (middle panel) and after orthotopic implantation (right panel).
(TIF)

## References

[1] Gross RE (1951) Treatment of certain aortic coarctations by homologous grafts; a report of nineteen cases. Ann Surg 134: 753–768. 14878385 10.1097/00000658-195110000-00020 PMC1802983

[2] Voorhees AB Jr, Jaretzki A 3rd, Blakemore AH (1952) The use of tubes constructed from vinyon "N" cloth in bridging arterial defects. Ann Surg 135: 332–336. 14903863 10.1097/00000658-195203000-00006 PMC1802338

[3] Kuzmik GA, Sang AX, Elefteriades JA (2012) Natural history of thoracic aortic aneurysms. J Vasc Surg 56: 565–571. 22840907 10.1016/j.jvs.2012.04.053

[4] Kamiya H, Hagl C, Kropivnitskaya I, Weidemann J, Kallenbach K, et al (2007) Quick proximal arch replacement with moderate hypothermic circulatory arrest. Ann Thorac Surg 83: 1055–1058. 17307459 10.1016/j.athoracsur.2006.09.085

[5] LeMaire SA, Price MD, Parenti JL, Johnson ML, Lay AD, et al (2011) Early outcomes after aortic arch replacement by using the Y-graft technique. Ann Thorac Surg 91: 700–707. 21352982 10.1016/j.athoracsur.2010.11.008

[6] Estrera AL, Miller CC, Lee TY, Shah P, Irani AD, et al (2010) Integrated cerebral perfusion for hypothermic circulatory arrest during transverse aortic arch repairs. Eur J Cardiothorac Surg 38: 293–298. 20304662 10.1016/j.ejcts.2010.02.021

[7] Milewski RK, Pacini D, Moser GW, Moeller P, Cowie D, et al (2010) Retrograde and antegrade cerebral perfusion: results in short elective arch reconstructive times. Ann Thorac Surg 89: 1448–1457. 20417760 10.1016/j.athoracsur.2010.01.056

[8] Dobson G, Flewitt J, Tyberg JV, Moore R, Karamanoglu M (2006) Endografting of the descending thoracic aorta increases ascending aortic input impedance and attenuates pressure transmission in dogs. Eur J Vasc Endovasc Surg 32: 129–135. 16564712 10.1016/j.ejvs.2006.01.020

[9] Kim SY, Hinkamp TJ, Jacobs WR, Lichtenberg RC, Posniak H, et al (1995) Effect of an inelastic aortic synthetic vascular graft on exercise hemodynamics. Ann Thorac Surg 59: 981–989. 7695428 10.1016/0003-4975(95)00068-v

[10] Scharfschwerdt M, Sievers HH, Greggersen J, Hanke T, Misfeld M (2007) Prosthetic replacement of the ascending aorta increases wall tension in the residual aorta. Ann Thorac Surg 83: 954–957. 17307439 10.1016/j.athoracsur.2006.10.056

[11] Bauernschmitt R, Schulz S, Schwarzhaupt A, Kiencke U, Vahl CF, et al (1999) Simulation of arterial hemodynamics after partial prosthetic replacement of the aorta. Ann Thorac Surg 67: 676–682. 10215210 10.1016/s0003-4975(99)00046-6

[12] Schulz S, Bauernschmitt R, Schwarzhaupt A, Vahl CF, Kiencke U (1997) Hemodynamic consequences of replacing the aorta by vascular grafts simulated in a mathematical model. Biomed Sci Instrum 34: 263–268. 9603050

[13] Mitsui T, Maeta H, Fukuda I, Ijima H, Okamura K, et al (1986) Left ventricular hypertrophy due to aortic bypass grafting with a long prosthesis. J Cardiovasc Surg (Torino) 27: 201–206. 2936751

[14] Maeta H, Hori M (1985) Effects of a lack of aortic "Windkessel" properties on the left ventricle. Jpn Circ J 49: 232–237. 2857795 10.1253/jcj.49.232

[15] Weston MW, Rhee K, Tarbell JM (1996) Compliance and diameter mismatch affect the wall shear rate distribution near an end-to-end anastomosis. J Biomech 29: 187–198. 8849812 10.1016/0021-9290(95)00028-3

[16] Abbott WM, Megerman J, Hasson JE, L'Italien G, Warnock DF (1987) Effect of compliance mismatch on vascular graft patency. J Vasc Surg 5: 376–382. 3102762

[17] Mehigan DG, Fitzpatrick B, Browne HI, Bouchier-Hayes DJ (1985) Is compliance mismatch the major cause of anastomotic arterial aneurysm? Analysis of 42 cases. J Cardiovasc Surg (Torino) 26: 147–150. 3156861

[18] Fabjan E, Hulzebos E, Mennes W, Piersma AH (2006) A category approach for reproductive effects of phthalates. Crit Rev Toxicol 36: 695–726. 17050082 10.1080/10408440600894914

[19] Engel SM, Miodovnik A, Canfield RL, Zhu C, Silva MJ, et al (2010) Prenatal phthalate exposure is associated with childhood behaviour and executive functioning. Environ Health Perspect 118: 565–571. 20106747 10.1289/ehp.0901470 PMC2854736

[20] Ito RK, Rosenblatt MS, Contreras MA, Brophy CM, LoGerfo FW (1990) Monitoring platelet interactions with prosthetic graft implants in a canine model. ASAIO Trans 36: M175–178. 2147555

[21] Weymann A, Dohmen PM, Grubitzsch H, Dushe S, Holinski S, et al (2010) Clinical experience with expanded use of the Ross procedure: a paradigm shift? J Heart Valve Dis 19: 279–285. 20583389

[22] Weymann A, Schmack B, Okada T, Soós P, Istók R, et al (2013) Reendothelialization of Human Heart Valve Neoscaffolds Using Umbilical Cord-Derived Endothelial Cells. Circ J 77: 207–216. 23001070 10.1253/circj.cj-12-0540

[23] Weymann A, Loganathan S, Takahashi H, Schies C, Claus B, et al (2011) Development and evaluation of a perfusion decellularization porcine heart model—generation of 3-dimensional myocardial neoscaffolds. Circ J 75: 852–860. 21301134 10.1253/circj.cj-10-0717

[24] Korkmaz S, Radovits T, Barnucz E, Hirschberg K, Neugebauer P, et al (2009) Pharmacological activation of soluble guanylate cyclase protects the heart against ischemic injury. Circulation 120: 677–686. 19667237 10.1161/CIRCULATIONAHA.109.870774

[25] Szabó G, Buhmann V, Graf A, Melnitschuk S, Bährle S, et al (2003) Ventricular energetics after the Fontan operation: contractility-afterload mismatch. J Thorac Cardiovasc Surg 125: 1061–1069. 12771880 10.1067/mtc.2003.405

[26] Rourke M, Taylor MG (1967) Input impedance of the systemic circulation. Circ Res 19: 365–380. 10.1161/01.res.20.4.365 6025401

[27] Attinger EO, Anne A, McDonald DA (1996) Use of Fourier series for the analysis of biological systems. Biophys J 6: 291–304. 10.1016/S0006-3495(66)86657-2 PMC1367946 5962280

[28] Cho SW, Lim SH, Kim IK, Hong YS, Kim SS, et al (2005) Small-diameter blood vessels engineered with bone marrow-derived cells. Ann Surg 241: 506–515. 15729075 10.1097/01.sla.0000154268.12239.ed PMC1356991

[29] Mekkaoui C, Rolland PH, Friggi A, Rasigni M, Mesana TG (2003) Pressure-flow loops and instantaneous input impedance in the thoracic aorta: another way to assess the effect of aortic bypass graft implantation on myocardial, brain, and subdiaphragmatic perfusion. J Thorac Cardiovasc Surg 125: 699–710. 12658214 10.1067/mtc.2003.104

[30] Sunagawa K, Maughan D, Burkhoff D, Sagawa K (1983) Left ventricular interaction with arterial load studied in isolated canine ventricle. Am J Physiol 254: H773–780. 10.1152/ajpheart.1983.245.5.H773 6638199

[31] Gao Y, Zou XM, Wang WJ, Liu GW, Gu MN (2006) Experimental study of cerebral protection by retrograde vs selective antegrade cerebral perfusion during deep hypothermic circulatory arrest. Nan Fang Yi Ke Da Xue Xue Bao 26: 644–647. 16762873

