# Supplementary material for: Total Aortic Arch Replacement: Superior Ventriculo-Arterial Coupling with Decellularized Allografts Compared with Conventional Prostheses
Source: PLoS One. 2014 Jul 31;9(7):e103588. doi: 10.1371/journal.pone.0103588 (PMC4117632; doi:10.1371/journal.pone.0103588)
Supplement: Data S1 — Supplementary methods. Detailed description of the methods used in the study. (DOC) [file pone.0103588.s001.doc]

SUPPORTING INFORMATION FILE "DATA S1"

for " Total aortic arch replacement: Superior ventriculo-arterial coupling with decellularized allografts compared with conventional prostheses"

**1. Supplementary Methods**

**Histological Analysis**

Segments of decellularized aortic arch allografts and freshly harvested aortic arches (control) were fixed in 10% phosphate-buffered formalin and embedded in paraffin. Sections (5-μm) were stained with hematoxylin-eosin (HE) to determine if residual nuclear structures could be identified after decellularization. Masson’s trichrome staining was used to distinguish the cells from the surrounding connective tissue (Sigma-Aldrich, Germany), and the Movat’s pentachrome stain (Mastertechs, Lodi, CA, USA) was used to visualize different extracellular matrix (ECM) components such as collagen, elastin, and proteoglycans. Tissue sections were analyzed using routine bright-field microscopy and fluorescence microscopy (Olympus Optical Co, BX 51 and CKX 41 microscopes). Images were acquired with the CellA Soft Imaging System (Olympus Soft Imaging Solutions®, Germany).

**General Management and Surgical Preparation**

The dogs were premedicated with acepromazine (0.03mg/kg i.m.), anesthetized with pentobarbital (30mg/kg initial bolus and then 25mg/kg/h i.v.), ketamine (1mg/kg initial bolus and then 1.5mg/kg/h i.v.) and tramadol (100mg i.v. bolus), paralyzed with pancuronium bromide (0.1mg/kg as a bolus and then 0.2 mg/kg/h i.v.) and endotracheally intubated. The dogs were ventilated with a mixture of room air and O2 (FiO2=60%) at a frequency of 12-15/min and a tidal volume starting at 15ml/kg per minute. The settings were adjusted by maintaining arterial partial carbondioxide pressure levels between 35-40mmHg. The right femoral artery was cannulated for taking blood samples for the analysis of blood gases, electrolytes and pH, and parameters of blood coagulation. Basic intravenous volume substitution was carried out via the right femoral vein with Ringer´s solution at a rate of 1ml/min/kg. If necessary, the rate of volume substitution was modified according to the continuously controlled input-output balance in order to maintain hemodynamic stability. According to the values of potassium, bicarbonate and base excess, substitution included administration of potassium chloride and sodium bicarbonate (8.4%). Body temperature was monitored continuously. Arterial pressure was monitored with 6F Millar pressure catheter inserted into the abdominal aorta via the left femoral artery (Millar Instruments, Houston, TX, USA).

**Hemodynamic measurements and analysis**

Hemodynamic measurements were performed at baseline (before starting CPB) and after total aortic arch replacement (15min after weaning from CPB).

For the period of the hemodynamic measurements, the 6F Millar pressure catheter inserted for arterial pressure monitoring, was advanced from the abdominal into the descending thoracic aorta for recording aortic pressure close to the distal end of the aortic arch. Two perivascular ultrasonic flow probes (Transonic Systems Inc., Ithaca, NY, USA) were attached to the ascending and to the descending thoracic aorta, close to the proximal and distal end of the aortic arch, respectively, for recording aortic blood flow at these two sites.

Left ventricular (LV) pressures and volumes were measured by a combined 6F Millar pressure-volume (P-V) conductance catheter with 6mm spacing (SPC-561-1, Millar Instruments), which was inserted into the left ventricle via the apex. Signals from both pressure catheters, both flow probes and from the pressure-conductance catheter were recorded at a sampling rate of 1,000 samples/s using a P-V conductance system (MPVS-Ultra, Millar Instruments) connected to the PowerLab 16/30 data acquisition system (AD Instruments, Colorado Springs, CO, USA), stored, and displayed on a personal computer by the LabChart Pro V7 Software System (AD Instruments). Cardiac output (CO) and stroke volume (SV) were calculated from the integrated flow signal at the ascending aorta and were used to calibrate the volume signal from the conductance catheter. Parallel conductance was estimated by rapid injection of 1ml of hypertonic saline into the left atrium. LV P-V loops were constructed on-line. Vena cava occlusions were performed to obtain a series of loops for assessment of LV end-systolic P-V relationships (ESPVR) [1].

Time constant of LV pressure decay (τ) was calculated according to the Weiss method. Total peripheral resistance (TPR) was calculated as the quotient of mean arterial pressure (MAP) and CO (TPR=MAP/CO). Stroke work (SW) was calculated as the area within the P-V loop, pressure-volume area (PVA) was calculated as the area circumscribed by the end-systolic pressure-volume line, the end-diastolic pressure-volume relation curve, and the systolic pressure-volume trajectory. The SW/PVA ratio was defined as mechanical efficiency (Eff). Cardiac index (CI), total peripheral resistance index (TPRI) and stroke work index (SWI) were expressed by normalizing CO, TPR and SW to the body weight of the animal, respectively.

Arterial elastance (Ea) was calculated as the quotient of end-systolic pressure (Pes) and SV (Ea=Pes/SV). End-systolic elastance (Ees) was determined as the slope of the ESPVR. Ventriculoarterial coupling (VAC) was described by the quotient of Ea and Ees (VAC=Ea/Ees) [2].

Because flow through the cardiovascular system is pulsatile, conventional analysis excludes the significant contribution of pulsatile flow to the understanding of systemic hemodynamics. This leads to an underestimation of ventricular energy requirements. Therefore, with the help of a commercially available software (Origin 7G; OriginLab, Northampton, MA, USA) we calculated the vascular impedance spectrum through Fourier transformation [3,4]. The concept of Fourier analysis is based on the general principle that periodic waves can be mathematically expressed as a sum of a series of pure sinusoidal harmonics. This Fourier series contains a zero frequency (mean) and oscillatory harmonic frequencies that are integer multiples of the original periodic wave form. At each individual harmonic, division of the pressure amplitude by the flow amplitude allowed calculation of the oscillatory counterpart of resistance, or impedance, at that respective harmonic. Input impedance (RIN) was the impedance calculated at the zero harmonic and is a measure of resistance to mean systemic blood flow. Characteristic impedance (Z) was estimated from the following 6 harmonics and is a measure of resistance to pulsatile blood flow.

**2. References to Supplementary Methods**

1. Korkmaz S, Radovits T, Barnucz E, Hirschberg K, Neugebauer P, et al. (2009) Pharmacological activation of soluble guanylate cyclase protects the heart against ischemic injury. Circulation 120: 677-686.

2. Szabó G, Buhmann V, Graf A, Melnitschuk S, Bährle S, et al. (2003) Ventricular energetics after the Fontan operation: contractility-afterload mismatch. J Thorac Cardiovasc Surg 125: 1061-1069.

3. Rourke M, Taylor MG. (1967) Input impedance of the systemic circulation. Circ Res 19: 365-380.

4. Attinger EO, Anne A, McDonald DA. (1996) Use of Fourier series for the analysis of biological systems. Biophys J 6: 291-304.
