# Supplementary figures and images for: Total Aortic Arch Replacement: Superior Ventriculo-Arterial Coupling with Decellularized Allografts Compared with Conventional Prostheses
Source: PLoS One. 2014 Jul 31;9(7):e103588. doi: 10.1371/journal.pone.0103588 (PMC4117632; doi:10.1371/journal.pone.0103588)

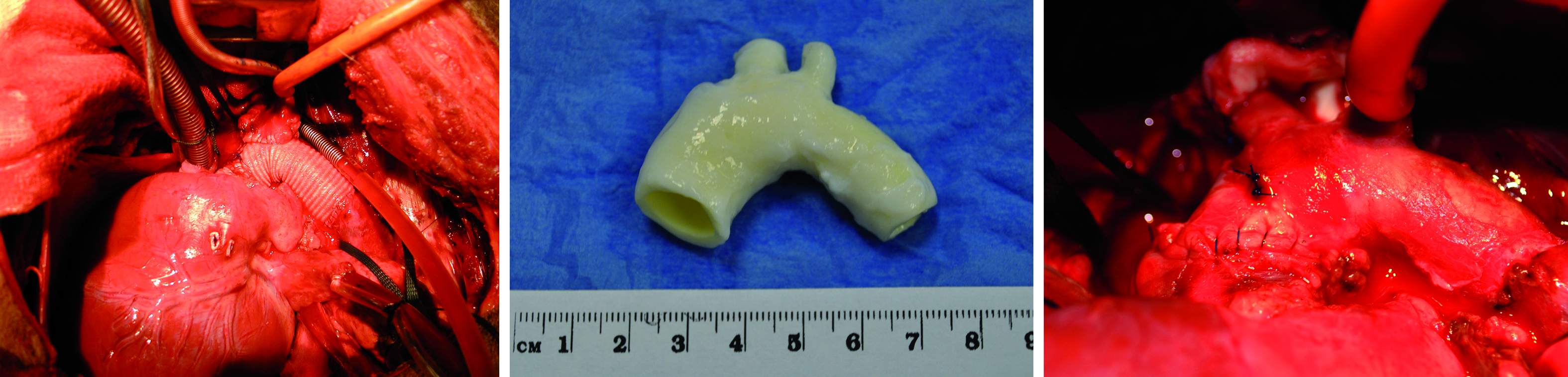

Supplement: Figure S1 — Photographs of the prosthesis and decellularized allografts. Representative images of macroscopic appearance of an implanted conventional prosthesis (left panel), decellularized aortic arch allograft before (middle panel) and after orthotopic implantation (right panel). (TIF) [file pone.0103588.s002.tif]
